# Target 5000: a standardized all-Ireland pathway for the diagnosis and management of inherited retinal degenerations

**DOI:** 10.1186/s13023-021-01841-1

**Published:** 2021-05-05

**Authors:** Kirk A. J. Stephenson, Julia Zhu, Niamh Wynne, Adrian Dockery, Rebecca M. Cairns, Emma Duignan, Laura Whelan, Conor P. Malone, Hilary Dempsey, Karen Collins, Shana Routledge, Rajiv Pandey, Elaine Crossan, Jacqueline Turner, James J. O’Byrne, Laura Brady, Giuliana Silvestri, Paul F. Kenna, G. Jane Farrar, David J. Keegan

**Affiliations:** 1 Mater Clinical Ophthalmic Genetics Unit, Mater Misericordiae University Hospital, Dublin, Ireland; 2 The Research Foundation, Royal Victoria Eye and Ear Hospital, Dublin, Ireland; 3 Ocular Genetics Unit, Smurfit Institute of Genetics, Trinity College Dublin, Dublin, Ireland; 4 Belfast Health and Social Care Trust Hospitals, Belfast, Northern Ireland; 5 National Council for the Blind of Ireland, Whitworth Road, Dublin 9, Ireland; 6 Fighting Blindness Ireland, Ely Place, Dublin 2, Ireland

**Keywords:** Inherited retinal degenerations, Retinal dystrophy, Ocular genetics, Genetic diagnosis, Clinical diagnostic algorithm, Public and patient involvement

## Abstract

**Introduction:**

Inherited retinal degenerations (IRD) are rare genetic disorders with > 300 known genetic loci, manifesting variably progressive visual dysfunction. IRDs were historically underserved due to lack of effective interventions. Many novel therapies will require accurate diagnosis (phenotype and genotype), thus an efficient and effective pathway for assessment and management is required.

**Methods:**

Using surveys of existing practice patterns and advice from international experts, an all-Ireland IRD service (Target 5000) was designed. Detailed phenotyping was followed by next generation genetic sequencing in both a research and accredited laboratory. Unresolved pedigrees underwent further studies (whole gene/whole exome/whole genome sequencing). Novel variants were interrogated for pathogenicity (cascade screening, in silico analysis, functional studies). A multidisciplinary team (MDT; ophthalmologists, physicians, geneticists, genetic counsellors) reconciled phenotype with genotype. A bespoke care plan was created for each patient comprising supports, existing interventions, and novel therapies/clinical trials.

**Results and discussion:**

Prior to Target 5000, a significant cohort of patients were not engaged with healthcare/support services due to lack of effective interventions. Pathogenic or likely pathogenic variants in IRD-associated genes were detected in 62.3%, with 11.6% having variants of unknown significance. The genotyping arm of Target 5000 allowed a 42.73% cost saving over independent testing, plus the value of MDT expertise/processing. Partial funding has transferred from charitable sources to government resources.

**Conclusion:**

Target 5000 demonstrates efficacious and efficient clinical/genetic diagnosis, while discovering novel IRD-implicated genes/variants and investigating mechanisms of disease and avenues of intervention. This model could be used to develop similar IRD programmes in small/medium-sized nations.

**Supplementary Information:**

The online version contains supplementary material available at 10.1186/s13023-021-01841-1.

## Introduction

Inherited retinal degenerations (IRDs) are a group of rare eye diseases that result in variable and progressive vision loss. IRDs have a heterogeneous phenotype and genotype with over 300 causal genes identified to date [1], the first IRD-implicated gene (*RHO*) having been identified by co-authors in Dublin [2, 3]. Developed with support from international colleagues [4–7], a systematic all-Ireland plan was proposed for the evaluation and management of IRDs. The goal of this programme was to coordinate medical, academic and financial resources to achieve collaborative validated clinical and genetic diagnosis, to introduce relevant supports and to achieve preparedness for clinical trials and novel therapies both in Ireland [8] and internationally [9–12].

Due to the systematic management of more common eye conditions (i.e., diabetic retinopathy (DR) screening programmes), IRDs have surpassed DR as the leading cause of blind registration in adults < 64 years old in England and Wales [13]. Treatment algorithms have been established for more prevalent eye diseases (e.g. DR, 1.7% of global population [14], age-related macular degeneration, 7.2% of Irish population > 50 years [15]), while IRDs do not have a standardized approach. Despite IRDs being rare diseases, they accrue an €80 million per annum socioeconomic burden [16], with patients often seeking out and paying for expertise abroad at their own initiative. Ophthalmologists (and all eye care professionals) are obliged to advocate for the IRD population (3:10,000) by striving for a similar world-class standard of care with efficient use of available resources.

The ideal pathway is efficient (short turnaround time, i.e., avoiding prolonged delay and the consequent progression of retinal atrophy), accurate (correct clinical and genetic diagnosis) and effective (allowing access to relevant interventions, both supportive and disease-modifying). Combs et al. defined three main output requirements from an IRD focus group. The first was diagnosis and prognosis, allowing relevant family planning and targeting access to clinical research, trials, and treatments. The second requirement was psychological support (i.e., in adjusting to and coping with diagnosis) and the third was practical physical supports (e.g., financial, education, low vision). Respondents to this survey and a recent Irish study expressed dissatisfaction at the lack of integration between health and social care, which may explain lack of representative engagement between people with IRDs and health services [4, 16]. A set of core goals was distilled to best utilise the limited resources available for IRD management in Ireland:

1. Genetically resolve IRD pedigrees,
2. Act on modifiable disease,
3. Offer relevant supports, and
4. Provide access to clinical trials of novel therapeutics when appropriate.

**Fig. 1:**
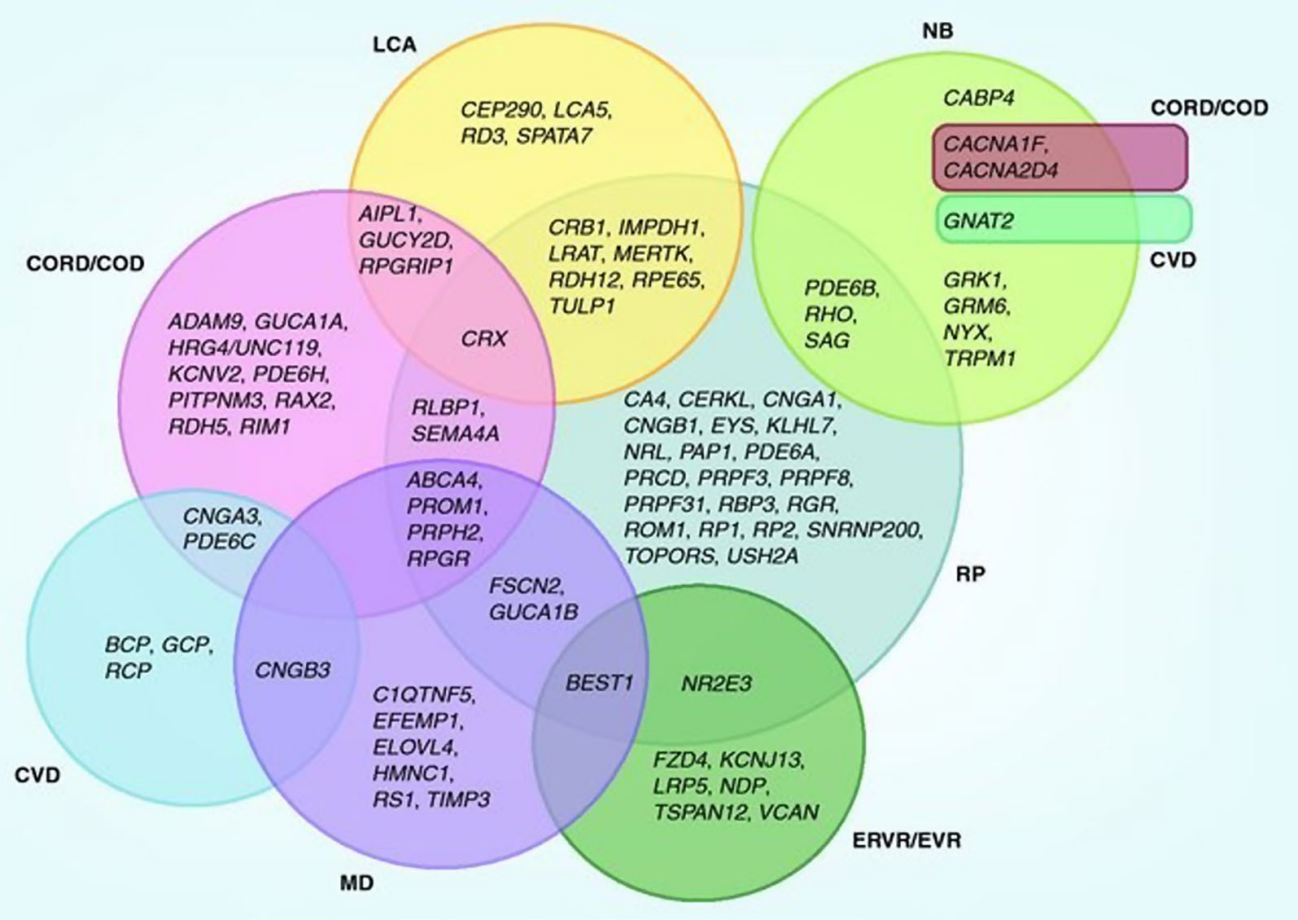
Reproduced with permission from Sutherland et al. [18]. This figure highlights the pleiotropic nature of genes implicated in IRDs with 1 gene potentially manifesting as multiple/overlapping phenotypes in different individuals. Likewise, a single phenotype may have multiple genetic aetiologies. (CORD/COD Cone-Rod Dystrophy/Cone Dystrophy, CVD colour vision defects, ERVR/EVR Erosive and exudative vitreoretinopathies, LCA Leber Congenital Amaurosis, MD macular dystrophy, NB night blindness, RP retinitis pigmentosa)

IRDs are often heterogeneous with a similar clinical phenotype caused by mutations in multiple possible genes (Fig. 1). Likewise, a single gene (e.g., *ABCA4* OMIM * 601691) may manifest as variable phenotypes (e.g., Cone-rod dystrophy-3, #604116; Fundus flavimaculatus #248200; Retinal dystrophy, early-onset severe, #248200; Retinitis pigmentosa-19, #601718; Stargardt disease-1, #248200; Macular degeneration, age-related-2, #153800). Genetic pleiotropy in IRDs (e.g. *USH2A* gene, *608400) may manifest with syndromic (Usher Syndrome type 2A, #276901) or non-syndromic (retinitis pigmentosa-39, #613809) features dependent on the position and deleteriousness of the variants within the gene [17]. Given the complexity of achieving an accurate diagnosis, a coordinated and qualified multidisciplinary team (MDT) approach was adopted to reach an evidence-based consensus for diagnosis and management of each case. An accurate genetic diagnosis is critical in coordinating career/family-planning choices, determining prognosis and accessing gene-specific therapies [18]. This requires detailed phenotyping (ocular and systemic), affordable and accurate clinically accredited genotyping, clinical genetics oversight, genetic counselling (GC), and connections to international networks for second opinions and access to clinical trials/treatments where available.

Adding to the complexity of accurate IRD diagnosis is the lack of agreed common terminology. Sergounotis et al. have contributed to clarification of this with the updated ocular human phenotype ontology and Orphanet ontology [19, 20]. Similarly, the American College of Medical Genetics (ACMG) grading is used to determine the likelihood of pathogenicity of detected genetic variants, these criteria being the current global standard [21]. International collaborations are required for second opinions from world-experts as well as access to clinical trials. Relationships in Europe were formalised with the founding of the European Reference Network for Rare Eye Disease (ERN-EYE) in which electronic IRD registers can be shared both for clinical and research purposes [22]. Using the above standardized clinical and genetic lexicon, meaningful outputs can be derived from this communal resource. The input of a physician/geneticist is critical, as the focus in syndromic disease is not only on visual prognosis but on detection and intervention for significant systemic disease (e.g., cardiac, metabolic, and renal disease).

For the first time, treatment is available for a small subset of IRDs, the first being approved for biallelic *RPE65* retinal degeneration in America (2017) and Europe (2018) [23] while therapies for some of the more frequent IRD phenotypes (e.g. choroideraemia, achromatopsia, X-linked retinitis pigmentosa, X-linked retinoschisis) are in advanced human clinical trials [9–12]. Thus, a major goal of programme participation was “clinical trial/therapy preparedness” (i.e., full clinical and genetic characterization to meet standard inclusion criteria for clinical trials where available). Although it began as a genetic research study, as determination of genotype has become mandatory for both precise diagnosis and eligibility for gene-specific trials of novel treatments, by necessity, Target 5000 has developed into a clinical programme.

This publication outlines the assessment of the pre-existing infrastructure for IRD management in the Republic of Ireland and the use of these data to develop and implement a purpose-built framework to assess and provide world-class care for IRDs on the island of Ireland (Northern Ireland and Republic of Ireland).

## Methods

In 2009, an expert working group of Irish IRD specialists (clinicians and scientists), with input from IRD patient groups, met to develop the blueprint of the Target 5000 programme which was to be run at 3 clinical sites (2 in Dublin, Ireland and 1 in Belfast, Northern Ireland) and 1 academic research laboratory (Dublin, Ireland). A consensus decision was made to start a common standardized programme for IRD assessment with a national register and individualized care plans. This genetic characterization study was approved by the institutional review boards of the participating hospitals (Mater Misericordiae University Hospital, Dublin, Ireland; Royal Victoria Eye and Ear Hospital, Dublin, Ireland; and later the Belfast Health and Social Care Trust Hospitals, Belfast, Northern Ireland) and university laboratory (Ocular Genetics Unit, Trinity College Dublin, Ireland). Funding was initially sourced from patient-initiated and other charitable sources (e.g., Health Research Board Ireland, Science Foundation Ireland, Health Research Charities Ireland (HRCI), Fighting Blindness Ireland. Programme funding has now been partly transferred to the national public health service (Health Service Executive, Ireland; HSE).

In 2014, a survey was conducted (Google Forms, Google LLC, Additional file 1) in 12 clinical ophthalmic sites in Ireland to assess the state of the pre-existing infrastructure for IRD assessment, to identify any areas of concern or omission and to inform the creation of a new national IRD pathway.

Patient recruitment was by referral from ophthalmologists, optometrists, and patient organizations to facilitate open access to participation. Clinical assessment was performed and, where a clinical diagnosis of IRD was suspected, patients were included in the genotyping and follow up arm of the study.

All patients were seen in IRD-specific subspecialty clinics. Meticulous phenotyping was performed including thorough history (ophthalmic, systemic, and family), comprehensive ophthalmic examination (visual acuity, dilated slit lamp biomicroscopy, intraocular pressure measurement), and ancillary clinical tests (multimodal retinal imaging (MMI), visual field assessment and ISCEV-standard electrophysiology). When available, historical data was included for analysis of progression. Initial genotyping was performed in the affiliated research-grade laboratory (Ocular Genetics Unit, Trinity College Dublin) by a panel-based target capture next generation sequencing (NGS) approach assessing all known IRD-associated genes as described in previous publications [24–27]. Genetically unresolved patients were further investigated with whole gene (i.e., phenotypes typically associated with 1 gene, e.g., Stargardt disease/ABCA4, Choroideraemia/CHM, etc.) [28], whole exome (i.e., reanalysis of unresolved pedigrees for new genes) or whole genome sequencing (i.e., phenotypes associated with many genotypes but not resolved with NGS/WES, e.g., retinitis pigmentosa) where appropriate [29]. Prime candidate variants from this initial assessment were confirmed by clinically accredited laboratories (Manchester Centre for Genomic Medicine or Blueprint Genetics, Helsinki Finland).

**Fig. 2:**
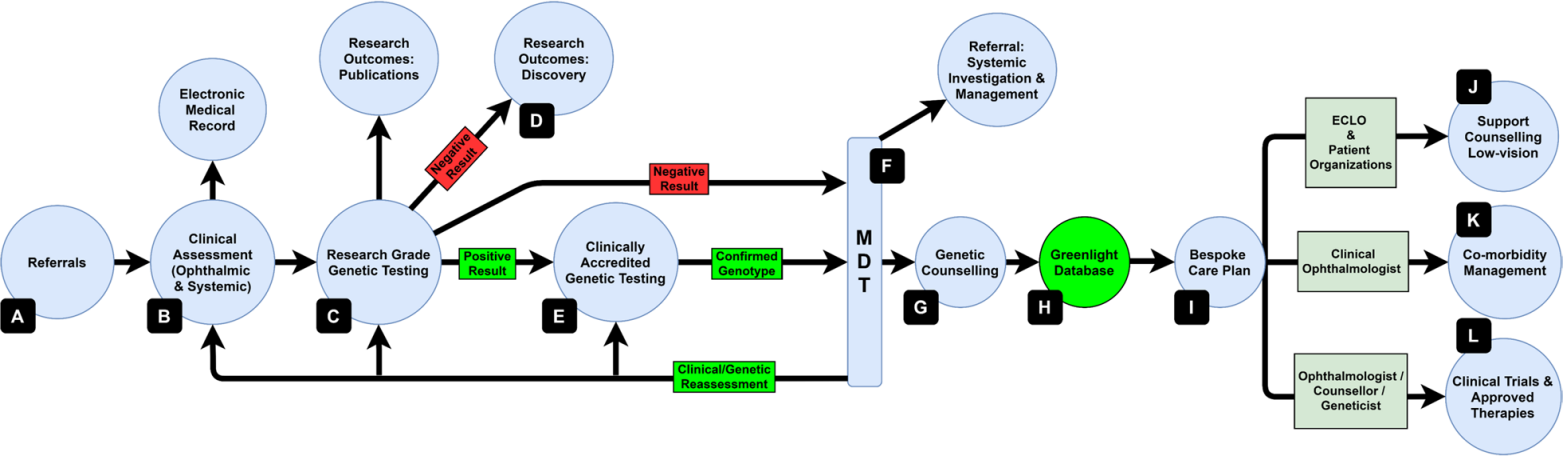
Schematic diagram of the Target 5000 IRD algorithm. The above figure illustrates the pathway employed by Target 5000 to phenotype and genotype patients with IRDs in Ireland. Following (A) referral and (B) clinical assessment, patients are (C) genotyped via an NGS panel-based approach of all known IRD-implicated genes at a research level. If the NGS panel test is negative they go onto (D) the discovery arm of Target 5000 including whole gene/exome/genome sequencing as appropriate. Novel variants are assessed with tools including cascade screening, in silico analysis and functional studies. If a candidate variant is identified, a biobanked sample is (E) sent to a clinically accredited laboratory for confirmatory testing. The phenotype is reconciled with the confirmed genotype via (F) clinical/genetic MDT discussion. If required, clinical and/or genetic reassessment or referral for systemic investigation/management is initiated at this stage. The clinical genetics team arrange a (G) genetic counselling session with the patient where the most appropriate interventions for each individual are discussed. Individuals meeting the prerequisites for this stage are eligible for entry on (H) the “Greenlight Database” signifying adequate phenotypic/genotypic characterization for (L) existing and novel (i.e., clinical trials) therapies. Underpinning the entire effort is the input of the ECLO who co-ordinates (J) supportive care (e.g., low vision aids, psychological counselling, and technology training). In parallel, the clinical team assess the (K) co-morbidities and introduce therapies such as cataract surgery or treatment for macular oedema. This multifaceted pathway results in (I) a bespoke care plan, which assesses all aspects of their eye condition, ensuring timely access to appropriate novel and established interventions

Confirmed variants were discussed using an MDT approach (ophthalmic clinicians, systemic physicians, geneticists, genetic counsellors). Published evidence, online databases (e.g. RetNet, ClinVar) and the ACMG criteria were used to confirm phenotype-genotype matches in line with the AAO guidelines [30] prior to GC sessions. Gene/condition-specific care plans were created for each genetic diagnosis (see below and Fig. 2).

## Results

The survey of the prior IRD standard of care confirmed the national absence of a standardized, coordinated approach to assessment. No formal interdepartmental register, nor electronic medical record, of patients and their diagnoses existed. Patients were often referred, at their own expense, to international experts for workup and management. Extrapolations from the survey of 12/16 (75%) responding ophthalmic centres revealed that 52% of the estimated IRD population were not being reviewed under the pre-existing conditions. Patients were assessed at IRD subspecialty clinics in only 33% of visits (vs 63% in the UK [5]), these centres being the founding collaborator Target 5000 clinical units. These subspecialist units had additional resources in terms of clinical IRD experience, greater time per patient (179% more time per patient visit, mean 47 versus 26.25 min, excluding time for investigations) and access to specific clinical investigations (e.g., MMI, electrophysiology), which were performed during the same visit rather than repeated journeys as in general clinics. Thirty-six per cent of attendees were from different counties than the clinical sites (1–4-h journey) and non-attendance at repeat appointments could be avoided by a comprehensive visit (i.e., full clinical assessment and investigations). These data show that fewer patients must be booked per clinic facilitating accurate clinical phenotyping and discussion with each patient as this has a knock-on effect on subsequent investigations (e.g., determining relevance of detected genetic variants), treatment options (i.e., clinical trial inclusion) and patient decisions.

Informed by these data, a new algorithm was created (Fig. 2) and delivered via a subspecialty service. Thus far, 1482 IRD patients (and unaffected relatives) of an estimated 2600 IRD patients in Ireland (Republic of Ireland and Northern Ireland) [16] have been clinically assessed/phenotyped (see B in Fig. 2). Research-grade NGS (C, Fig. 2) has been completed on 1004 individuals from 710 pedigrees; a causative genetic variant was detected in 495 pedigrees (69.7%), with a single allele variant detected in an additional 64 (9%) of clinically autosomal recessive (AR) pedigrees where an obligatory second pathogenic sequence variant could not be identified [26]. The remaining 478 phenotyped cases are pending shipment to the accredited genetic laboratory, delayed due to a combination of availability of HSE funding and COVID-19 restrictions. In general, the Target 5000 approach is ongoing clinical characterization of new IRD patients/pedigrees to front-load the service with phenotyped cases, which progress to genotyping as further funding becomes available. Gene discovery studies (D, Fig. 2, whole gene, whole exome, and whole genome sequencing) are being carried out on this cohort as well as in pedigrees where no variants in IRD-implicated genes were detected. Novel genetic variants were interrogated for pathogenicity via further studies (e.g., cascade screening, in silico analysis and functional studies). Confirmation of detected variants has been carried out via a clinically accredited genetics laboratory (E, Fig. 2) in 62.9% (440/700) of genetically resolved cases as of 30/06/2020 with the remainder pending. See Table 1.

**Table 1:** Completion rates of Target 5000 Algorithm Steps

	n =	% total	% category
Phenotyped (B)	1482	100	
Research NGS complete (C)	1004	67.7	
Positive research NGS	700	47.2	69.7
Negative research NGS	304	20.5	30.3
Accredited testing result (E)	440	29.7	62.9
Genetically eligible for trials	50	3.4	11.4

Stage indicated by letter in parentheses refers to Fig. 2

The MDT consensus for individual patients with a genetic outcome (from a subset of centres) was analysed (440 cases with clinically accredited genotype). ACMG pathogenicity scores for detected causative variants were grade 4 or 5 for 62.3% (n = 274), while 11.6% (n = 51) had at least 1 allele with a variant of unknown significance (VUS, ACMG grade 3). Further genetic confirmation (phase testing, carrier status) was requested in all AR cases. See Table 2 for more detail. MDT consensus has led to GC (either completed or pending) in 99% (n = 434/440) of cases. Of 440 IRD patients recruited at one of the Target 5000 clinical sites (MMUH), 50 patients (11.4%), thus far, are eligible for current therapies or active clinical trials (L, Fig. 2) (e.g., *RS1*, *USH2A*, *RPGR*, *CNGB3*, *CHM*, *RPE65*, *CEP290*).

**Table 2:** Outcomes of accredited genetic testing (n = 440); ACMG grading: Grade 5- pathogenic; Grade 4- likely pathogenic; Grade 3 -VUS

	n =	%
Grade 4 or 5	274	62.3
Grade 3 only	34	7.7
1 allele grade 3 and 1 allele grade 4/5 (AR disease)	17	3.9
No variants detected	19	4.3
Unclassified (regrading)	94	21.4
Benign	2	0.5
Total	440	

## Discussion

The rate of blind registration in the UK is 3% compared with 1.1% in Ireland, the age of whom was > 65 years in 66% and 72% of patients in the UK and Ireland respectively [31]. The mean age of all 1482 recruited patients in Target 5000 was 45.54 ± 18.67 years and 20.8% of these were registerable blind by visual acuity criteria (mean VA LogMAR 0.75 ± 0.87) not including those registerable by visual field constriction. Considering that IRDs are the leading cause of blindness in England and Wales in those < 64 years [13], IRDs are not proportionately represented and thus are not being adequately supported. Concurring with this, the internal survey mentioned above estimated 52% of the Irish IRD population was not being seen in eye clinics. This may reflect prior poor patient engagement due to frustration with the lack of accurate diagnoses and effective treatments [4]. As the ability of clinicians to positively impact visual prognosis improves, re-engagement with healthcare professionals becomes vital to keep abreast of relevant developments and access novel treatments.

Genetic testing in overseas laboratoriess was historically a major component of the cost involved in IRD assessment. Prior to Target 5000, this was not centralized and charges were on an individual basis from either the public departmental resources or private patient funds. One of the main goals of the Target 5000 pathway was to create an equitable standardized level of care for all service users with quality and cost benefits to the health service. Initial funding for Target 5000 was provided from multiple charitable and grant sources (see methods). Genotyping through a common pathway at a new high throughput single accredited laboratory has allowed for significant savings (50.8%, €1000 vs €1968 per test). Using an example of 1522 IRD-affected individuals [16], the cost of genetic testing for all patients and 1 relative would be €3.80 million and €2.18 million outside and inside the Target 5000 programme respectively, saving 42.73%. This estimate does not account for the cost of access to the IRD clinical and genetics testing setup and expertise, nor the ophthalmic genetics MDT, which is unavailable outside the programme. The panel-based NGS approach in a research-based academic laboratory with validation in an accredited laboratory adopted by Target 5000 accrued substantial cost savings [26]; however, due to limited financial, personnel and bioinformatics support inherent in research-based academic laboratories the interval to receive a genetic result was circa 18–24 months. This is due to additional workup including cascade analysis (clinically and genetically testing additional family members and confirming segregation of variants within the family), in silico analysis and functional studies (e.g., investigation of splicing of non-coding variants in the *ABCA4* gene) to explore the pathogenicity of novel variants in IRD-implicated genes. Much of this work has been undertaken at a research-grade level to date highlighting the diverse investigations required to genetically resolve IRDs, the cost of which is difficult to estimate. Delay has significantly reduced with an NGS panel-based genetic result available from a high throughput accredited commercial laboratory within 4 weeks. This enables early recognition of cases suitable for disease modifying therapies before further progressive retinal atrophy has developed, though the contribution of the in-house MDT in correlating genotype to the clinical phenotype remains a vital component of this process. The ongoing development of a domestic accredited genetic laboratory with further capacity and bioinformatics support could further reduce this cost per patient/pedigree. The outcomes of the programme so far have been used as evidence to secure further limited funding from the public health service (HSE Ireland). Given that the cost of whole genome sequencing (WGS), data analysis and storage will likely continue to reduce, it may be that the field will move towards this technique for the investigation of all IRD patients from the outset.

A comprehensive database of available support services was compiled to facilitate access for patients as appropriate (Additional file 2). An on-site eye clinic liaison officer (ECLO) was incorporated into the IRD clinic to assess areas of need and signpost available supports (e.g., social supports, housing, low vision aids, etc.), from first contact and modify them as necessary at subsequent visits.

An MDT approach was adopted, comprising ophthalmic clinicians, systemic physicians, geneticists, and genetic counsellors. This group met weekly to assess each case from a clinical and genetic perspective, with a national multicentre MDT monthly. The goals of the MDT were to:

1. Confirm a genotype/phenotype match,
2. Determine need for phase testing (all AR cases) or further family studies (clinical and/or genetic assessment), and
3. Assess the best available evidence-based interventions (supports and therapeutics).

Specific IRD clinics aim to provide goal-oriented patient interactions with specific progress-dependent goals at each visit. The first visit allows initial phenotyping and DNA sampling for genetic analysis, while early ECLO involvement initiates relevant supports. Affected and unaffected relative involvement can be sought at or following the initial visit. Subsequent visits assess subjective/objective progression, monitor treatment response (e.g., macular oedema, cataract) and update ECLO inputs. When a validated genotype becomes available, following MDT discussion of each case, GC can be performed with the patient (± family). At this stage, a bespoke care plan (curated by the MDT and modified for relevance by an IRD patient focus group) is introduced to highlight relevant areas of intervention (Zhu et al., in prep). As 71.2% of genetically diagnosed IRDs are due to pathogenic variants in 20 genes [32], a small number of care plans apply to the majority of pedigrees, while care plans for less common phenotypes/genotypes can be created and updated as required.

## Conclusion

The development of the Target 5000 pathway has allowed timely and accurate MDT-validated clinical and genetic diagnoses for patients with known pathogenic variants while enabling detection and exploration of pathogenicity for novel variants, thus increasing global understanding in this field (i.e., RetNet, ClinVar, etc.). A coordinated register of the clinically and genetically characterized IRD population becomes increasingly relevant as the scientific and medical community better understand the mechanisms of disease and potential avenues of intervention for these rare diseases. Findings from this pathway can be distilled into accessible and patient-friendly bespoke care plans which guide actionable outcomes for the individual. In addition to streamlined access to supports and standard clinical care (e.g., cataract, cystoid macular lesions, refractive correction), clinical trials and novel therapies are within reach via pharmaceutical industry directly or via the link to ERN-EYE, a first for IRD patients in Ireland [8]. The Target 5000 template could be used to guide development of IRD programmes in small to medium-sized countries in concordance with international best practice (Table 3).

**Table 3:** Recommendations for developing a national IRD programme

Identify national centres of ophthalmic expertise that are staffed and resourced to see patients with IRDs	
Engage and partner with support agencies and patient organisations	
Concentrate clinical genetics and support services at these sites	
Encourage referrals of all diagnosed or suspected IRD cases to these centres. Follow up can be at regional centres with appropriate clinical testing capability	
Have a robust pathway for funded genetic testing and resolution of contentious variants (via MDT and partnerships with other international laboratories)	
Routinely use and regularly update variant classification (e.g., in silico and functional analysis for novel variants) and collaborative databases (e.g., ClinVar, RetNet)	
Become members of international alliances (e.g., ERN-EYE) to share and gain knowledge and expertise of rare eye disease	
Establish therapeutic pathways nationally and internationally	
Create bespoke care plans for each patient/pedigree	
Audit outcomes for patients	

## Supplementary Information

**Additional file 1.** Survey and analysis of pre-existing infrastructure for assessment and management of IRDs in the Republic of Ireland.

**Additional file 2.** Services provided by vision impairment support groups/organizations in the Republic of Ireland and other western countries.

## Data Availability

All data generated or analysed during this study are included in this published article and its figures and tables.

## Abbreviations

ACMG: American College of Medical Genetics
AR: Autosomal recessive
DR: Diabetic retinopathy
ECLO: Eye clinic liaison officer
ERN-EYE: European Reference Network for Rare Eye Disease
GC: Genetic counselling
HRCI: Health Research Charities Ireland
HSE: Health Service Executive (Ireland)
IRD: Inherited retinal degeneration
ISCEV: International Society for Clinical Electrophysiology of Vision
MDT: Multidisciplinary team
MMI: Multimodal imaging
NGS: Next generation sequencing
VUS: Variant of unknown significance
WGS: Whole genome sequencing
