# Supplementary material for: Target 5000: a standardized all-Ireland pathway for the diagnosis and management of inherited retinal degenerations
Source: Orphanet J Rare Dis. 2021 May 5;16:200. doi: 10.1186/s13023-021-01841-1 (PMC8097252; doi:10.1186/s13023-021-01841-1)
Supplement: Supplementary file 1 — Additional file 1. Survey and analysis of pre-existing infrastructure for assessment and management of IRDs in the Republic of Ireland. [file 13023_2021_1841_MOESM1_ESM.pdf]

Chapter 3: Survey of Current IRD Services in Ireland

Inherited Retinal Dystrophy (IRD) National Care Plan Assessment

* 1. On behalf of which hospital/clinical centre are you completing this survey?

[ ]

* 2. At your centre, do you see IRD patients in a dedicated IRD clinic or as part of your regular clinics?

- ○ Dedicated IRD clinic
- ○ Regular Ophthalmology Clinic

* 3. Where do you receive IRD referrals from? (select all that apply)

- ☐ Ophthalmology Clinics
- ☐ Private Ophthalmologists
- ☐ General Practitioners
- ☐ Optometry practices
- ☐ General medical clinics
- ☐ General paediatric clinics
- ☐ Genetics Clinics
- ☐ Other (please specify)

[ ]

* 4. How many NEW IRD patients do you see per month (average)?

- ○ None
- ○ 1-5
- ○ 6-10
- ○ 11-15
- ○ >15

5. How many RETURN IRD patients do you see per month (average)?

- ○ None
- ○ 1-5
- ○ 6-10
- ○ 11-15
- ○ >15

* 6. Which staff are available to see IRD patients on the day of their clinic visit? (select all that apply)

- ☐ Consultant Ophthalmologist
- ☐ Ophthalmology NCHD
- ☐ Clinical Geneticist
- ☐ Genetic counsellor
- ☐ Optometrist / low vision specialist
- ☐ Liason officer
- ☐ Social worker
- ☐ General clinic administrative staff
- ☐ Specialized IRD clinic administrative staff

* 7. How much time is given for an IRD patient's FIRST appointment (single patient)?

- ○ <15 minutes
- ○ 15-30 minutes
- ○ 30-60 minutes
- ○ >60 minutes
- ○ Other (please specify)

* 8. Which of the following do you perform at the first clinic visit for an IRD patient? (select all that apply)

- ☐ Give a provisional diagnosis
- ☐ Explain the prognosis
- ☐ Explain current management options (including disease modifying treatments and low vision aids)
- ☐ Perform clinical tests (e.g. imaging, visual fields)
- ☐ Request genetic testing

* 9. What tests do you routinely order for IRD patients seen in your clinics? (select all that apply)

- [ ] Electrophysiology
- [ ] Visual Fields
- [ ] Colour Fundus Photography
- [ ] Fundus Autofluorescence
- [ ] Optical Coherence Tomography (OCT)
- [ ] Genetic testing

Other (please specify)

10. Are you aware of the Target 5000 National Inherited Retinal Dystrophy project?

- [ ] Yes
- [ ] No

* 11. What management plan is implemented after the first patient visit?

- [ ] Referral to Target 5000 study (RVEEH or MMUH)
- [ ] Referral to local specialist ophthalmologist
- [ ] Genetic testing offerred
- [ ] Actively seek clinical trial entry for IRD patients
- [ ] No treatment/advice offered
- [ ] Other (please specify)

* 12. What are your indications for requesting genetic testing? (select all the apply)

- [ ] Patient request
- [ ] Pre-natal diagnosis (patient or partner)
- [ ] Confirm or clarify clinical diagnosis
- [ ] Research purposes

Other (please specify)

* 13. If a summary letter/correspondence is sent out, to whom is this letter sent?

- ◯ General practitioner
- ◯ Referring source (ophthalmologist/optometrist/physician)
- ◯ Patient
- ◯ Other (please specify)

14. Is this letter a generic letter or does it contain specific patient details?

- ◯ Generic letter
- ◯ Detailed unique letter

* 15. If you see IRD patients back in clinic, what is the purpose of the return appointment?

- ☐ To give diagnosis / genetic results
- ☐ To repeat tests and monitor for progression
- ☐ They are seen at the patient's request

16. If you would like to make any further comments on the assessment & management of IRD patients in your centre, please do so below. Thank you for your participation in this study.

3.1 Introduction

Assessment of a person with IRD requires a detailed history, examination and ancillary tests (see Chapter 2: Methods). It is the opinion of the working group of this study that this should be performed in centres of excellence for the specialty. These centres allow the concentration of resources (personnel and equipment) achieving a timely diagnosis, preventing misdiagnosis and centralising treatments and support services.

At present, IRD assessment and care is provided via multiple divided centres which provide general ophthalmology services. To assess whether adequate provision of care was in place with the current system, a survey was carried out. The format and structure were guided by a UK study of ophthalmic hospitals and genetic services for inherited retinal dystrophies in the NHS[56].

The goals of this survey were to ascertain:

1. The volume of patients with IRD attending Irish centres.
2. The availability of appropriate staff (medical, nursing, technical, counselling, NCBI)
3. Access to appropriate investigations (e.g. AF, OCT, VF, electrophysiology, genetic testing)
4. The current practice regarding management of IRDs

3.2 Methods

Clinical centres were selected to take part in the study on the basis of geographic distribution. Ten public hospitals (Figure 1, Table 1) and six private ophthalmology centres with known involvement in IRD care were invited to take part in the survey via monthly email invitation over a 3-month period.

A structured survey (Appendix 2) was sent by email to the head medical retinal specialist ophthalmologist at each unit. The survey was constructed via Google Forms survey creator (Google Inc. California, USA). Questions were in multiple choice format with room for free text commentary. The option was provided for further discussion via telephone conversation.

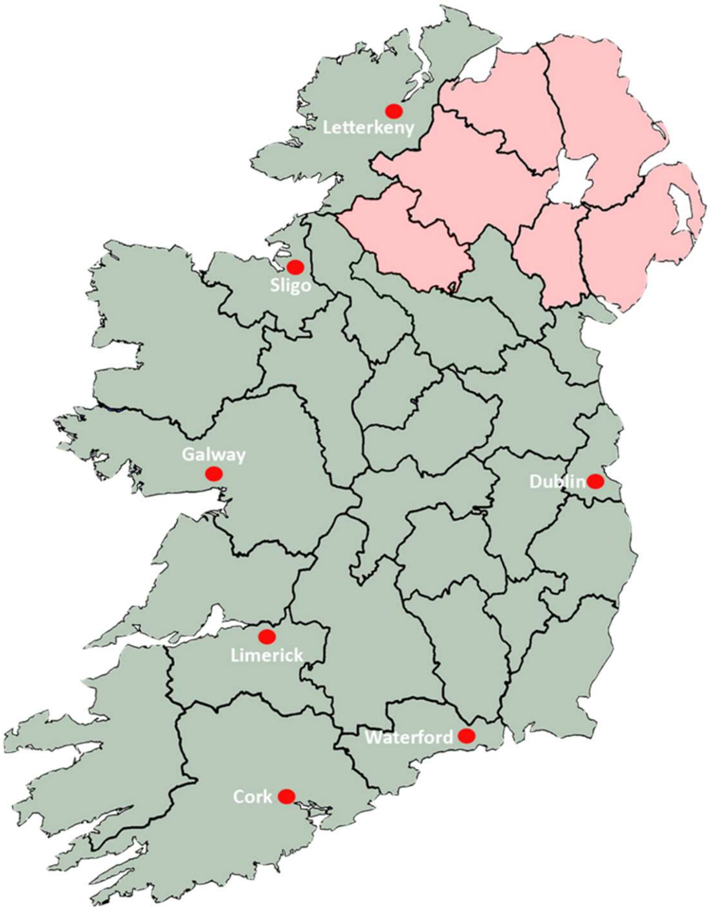

Figure 3.1. Map of Ireland demonstrating geographic distribution of public hospitals which participated in this survey. Centres surveyed were within the Republic of Ireland (i.e. not including Northern Ireland, though some families from the Republic may be seen by specialists in NI).

Specialist
MMUH
RVEEH
TSH
Crumlin
General Public
Waterford
Cork
Limerick
Galway
Sligo
Letterkenny
General Private
Galway Bons
Whitfield

Table 3.1 List of Participating Clinical Sites. This is divided into specialist vs general and private vs public.

Responses were collected digitally via Google Forms. The survey questions can be seen in Appendix 2. Topics covered included:

1. Type of clinic (i.e. general vs subspecialty)
2. Sources of IRD referral
3. Caseload of monthly new and return patients in set ranges (e.g. 1-5, 6-10, 10-15, >15)
4. Staff available (selection and free-text available)
5. Mean time allocated per clinic visit
6. Actions taken at the 1st clinic visit
7. Which clinical/laboratory tests were ordered
8. What plan, if any, was put into place
9. Availability of on-site low vision services
10. Indications for genetic testing (i.e. clinical trial entry, clarification of diagnosis)
11. Presence of absence of summary letter to GP
12. Free comment section

New patients were defined as people affected by IRD that were seen for the first time in the clinic. This was regardless of previous attendances at other centres within

Ireland or abroad. Review patients were defined as those that had been seen in a given ophthalmology centre and were being reviewed again in the same centre for further testing, treatment or monitoring for progression.

3.3 Results

The survey was conducted from July to September 2016. Twelve of 16 (75%) centres responded including the 2 centres involved in the Irish national IRD study (Target 5000), 2 (of 2, 100%) paediatric ophthalmology centres, 6 (of 6, 100%) regional public hospitals with adult and paediatric ophthalmology services and 2 (of 6, 33%) private ophthalmic practices from separate provinces. In the public system, this represents an even distribution of catchment areas through the country. The two Target 5000 centres have no catchment area restrictions and thus see patients from all areas of Ireland. Eleven responses (92%) were made via the Google Forms online interface. One response (8%) was documented via phone interview.

3.3.1 Volume of IRD Patients seen

Responses from all 12 centres revealed a monthly mean attendance of 3.96 new IRD patients and 4.63 review patients per centre. There were 47.5 new and 55.5 review patients seen per month which extrapolates to 570 new and 666 review patients (total) per year across all centres surveyed. The current population of Ireland is 4.7 million[34]. Extrapolating from international prevalence rates for IRD (approximately 1.75:3000 population[57]), there are an estimated 2741 people with IRD in Ireland. There is a definite shortfall in that 56% of the estimated IRD population is not being seen on an annual basis. This does not account for all private ophthalmic follow up. Of the 570 new patients that were seen annually across all participating centres, 61% were seen in the 4 specialist centres. Of the remaining patients seen in regional centres, 68% were seen publicly.

New	Total	Avg
All Centres	47.5	3.96
Specialist Centres	29	7.25
Regional Centres (combined)	18.5	2.31
Regional Public	12.5	2.09
Regional Private	6	3

Table 3.2: New Patients by centre type. The left column of this table ("Total") shows the combined number of new IRD patients seen monthly by the centre groups as labelled in the individual rows. The mean number of new patients seen by an individual centre of each centre type is shown to the right column ("Avg").

Review	Total	Avg
All Centres	55.5	4.63
Specialist Centres	29	7.25
Regional Centres (combined)	26.5	3.31
Regional Public	15.5	2.58
Regional Private	11	5.5

Table 3.3 Review Patients by centre type. The left column of this table ("Total") shows the combined number of patients seen monthly by the centre groups as labelled in the individual rows. The mean number of patients seen by an individual centre of each centre type is shown to the right-hand column ("Avg").

Of the 666 review IRD patients seen annually, 52% were seen in the 4 specialist centres. Of the remaining patients seen in general centres, 58% were seen publicly. There may be some overlap in the figures described above (i.e. a single patient may have been seen in multiple centres), but due to the lack of a centralised registry, this cannot be clarified/confirmed with the data management system of the current care model.

3.3.2 Format of Doctor-Patient Interactions

Clinic Format

One third of clinics responded that they saw IRD patients in specialist clinics. These were the centres designated as 'specialist centres' in the data analysis. 75% (n=3) of these were ophthalmic centres with a special interest in inherited eye conditions, 2 of which were the 2 Target 5000 centres. The remaining centre was a genetic clinic that sees many types of heritable conditions, which does not have specific ophthalmic genetic clinics, but for this patient group, is considered a specialist clinic. The remaining centres saw IRD patients in mixed general ophthalmic clinics, with no special allocation for time or resources.

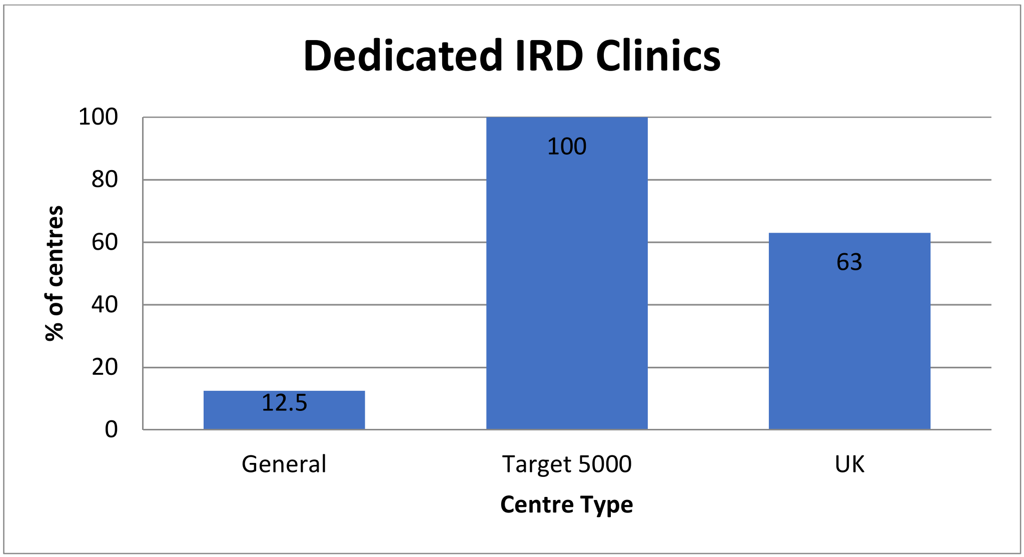

Figure 3.2 Percentage of Irish clinics with special focus on IRD compared with UK data. This is made possible by the small population of Ireland and the coordination between the 2 Target 5000 centres.

Centre Type	Mean Time Per Patient (minutes)
All Centres	35.63
Specialist Centres	46.88
Regional Centres (combined)	30
Regional Public	26.25
Regional Private	41.25

Table 3.4 Time allocation (mean) per patient visit by centre type.

Time allocation

The time allocated to each IRD patient visit was assessed as part of the survey. As portrayed in table 3.4, the mean time between all 12 centres surveyed was 35 minutes. Centres with specialist clinics had a 156% longer time per patient than the mean of all regional centres. Comparing regional centre subtypes, private centres had a mean time per patient 157% greater than public regional centres. Contrasting specialist centres with regional private centres showed specialist centres able to provide mean 114% greater time per patient.

Adequate time is required for the doctor-patient discussion of inherited retinal dystrophies. As much of the information is gene/condition specific, management, follow up and provision of results must be given in the proper setting with ample time for questions. Special consideration and clinic time allocation must be made for people with low vision and deafness (e.g. Usher Syndrome).

Available Staff

All surveyed centres had full administrative support for clinics. Eleven of the centres (92%) had a consultant ophthalmologist conducting the clinic. The remaining centre was run by a consultant clinical geneticist. Clinics in seven centres (58%) were attended by non-consultant hospital doctors (NCHDs) in addition to consultants.

Only 2 centres (17%), both from the specialist group, had a consultant level doctor with training in clinical genetics. A genetic counsellor was available to help return genetic results to patients in only one of the centres (8%). Low vision specialist optometrists were available on-site in 2 centres. An eye clinic liaison officer (ECLO) was available in 2 of the specialist centres (17%) and none of the general centres. On-site social workers were not present in any clinics surveyed.

Available Tests

Several clinical tests are relevant in the assessment of an IRD. The survey inquired about the availability and the regular use of these tests. The utilisation of these tests varied depending on whether the clinic was in a genetic or clinical ophthalmology centre.

All clinical ophthalmology centres that responded (11 sites, specialist and general) had access to and regularly used colour fundus photography (CP), optical coherence tomography (OCT) and visual field testing. Electrodiagnostic testing could be requested by all clinical centres but is only available on site in two centres. Nine (82%) of the clinical centres regularly utilised autofluorescence (AF) imaging. Six centres (50%) utilised genetic testing for IRDs: all four of the specialist centres and 2 (25%) of the general centres.

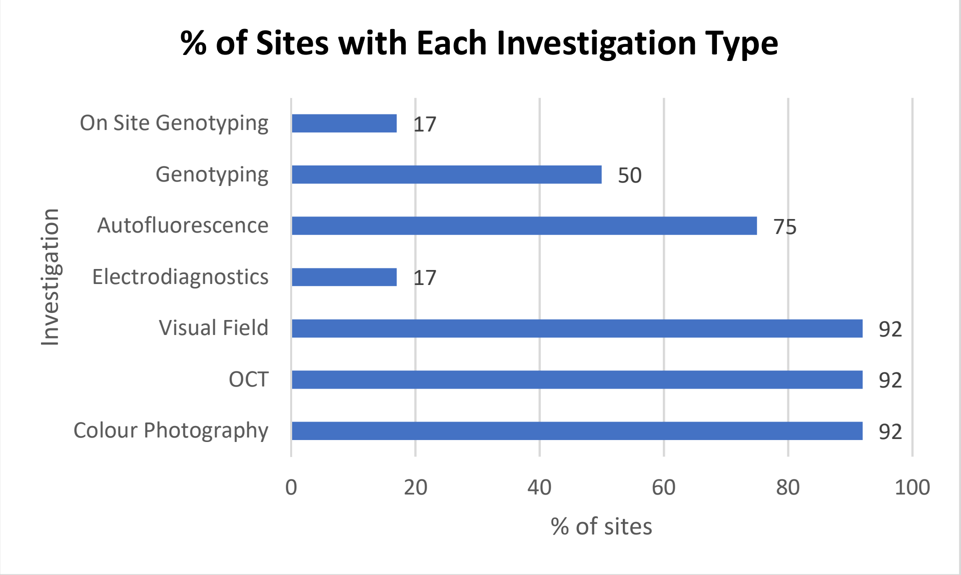

Figure 3.3 Percentage of centres with core investigation modalities.
