# Supplementary material for: Target 5000: a standardized all-Ireland pathway for the diagnosis and management of inherited retinal degenerations
Source: Orphanet J Rare Dis. 2021 May 5;16:200. doi: 10.1186/s13023-021-01841-1 (PMC8097252; doi:10.1186/s13023-021-01841-1)
Supplement: Supplementary file 2 — Additional file 2. Services provided by vision impairment support groups/organizations in the Republic of Ireland and other western countries. [file 13023_2021_1841_MOESM2_ESM.pdf]

	Available in Ireland													Available World Wide			
**Services/Products**	**NCBI**	**Arbour Hill Braille Unit**	**Assist Ireland**	**Child Vision**	**Fighting Blindness**	**Irish Guide Dogs**	**M.I.S.T.**	**National Assoc of Housing for the Visually Impaired**	**National League of the Blind**	**The Anne Sullivan Centre for the Deafblind**	**Visiting Teacher Service**	**Visually Impaired Computer Society of Ireland**	**Number of Service Providers in Ireland**	**United States**	**United Kingdom**	**Australia**	**Denmark**
Website Last Updated	22-Jun	NA	20-Apr	06-Jun	15-Jun	27-Jun	20-May	Dec-15	Dec-16	22-Mar	20-Mar	NA					
Early Intervention				✓						✓			2	✓	✓	✓	✓
Education Programmes				✓							✓		2	✓	✓	✓	✓
Rehabilitation Training/Therapies	✓			✓				✓			✓		4	✓	✓	✓	✓
Orientation and Mobility	✓			✓		✓							3	✓	✓	✓	✓
Braille Training	✓			✓				✓					3	✓	✓	✓	✓
Braille Production	✓	✓		✓									3		✓		
Daily Living	✓			✓		✓		✓		✓	✓		6	✓	✓	✓	✓
Residential Training/Living				✓		✓		✓		✓			4	✓	✓	✓	✓
Kitchen Skills	✓					✓		✓					3	✓	✓	✓	✓
Customer Service Skills	✓			✓		✓							3	✓	✓	✓	✓
Employment Service	✓			✓		✓							3	✓	✓	✓	✓
Money and Finance Services													0		✓		
Technology Help Groups	✓			✓	✓							✓	4	✓	✓	✓	✓
Community Integration Prgms	✓					✓		✓	✓				4	✓	✓	✓	✓
Activity Centers	✓			✓				✓	✓				4	✓	✓	✓	✓
Local Support Worker	✓							✓		✓			3	✓	✓	✓	✓
Counselling	✓				✓								2	✓	✓	✓	✓
Group Therapy					✓								1	✓	✓		✓
Support Groups	✓				✓		✓						3	✓	✓	✓	✓
Access Audit Services	✓					✓							2	✓	✓	✓	✓
Funding and Benefits									✓				1		✓		✓
Radio													0		✓	✓	✓
Library	✓			✓									2	✓	✓	✓	✓
Books	✓		✓										2	✓	✓	✓	✓
Braille Accessories	✓		✓										2	✓	✓	✓	✓
USB Players	✓		✓										2	✓	✓	✓	✓
DAISY and mp3 Players	✓		✓										2	✓	✓	✓	✓
Trascription Tools			✓										2	✓	✓	✓	✓
Talking Pens/Labels	✓		✓										2	✓	✓	✓	✓
Paper (pen and braille)	✓		✓										2	✓	✓	✓	✓
Writing Guides	✓		✓										2	✓	✓	✓	✓
Scented Pens	✓												1	✓	✓		
Bright Labelers	✓		✓										2	✓	✓	✓	✓
Bumpons	✓		✓										2	✓	✓	✓	✓
Magnifiers	✓		✓										2	✓	✓	✓	✓
Lamps with Magnifiers	✓		✓										2	✓	✓	✓	✓
Bulbs	✓												1	✓	✓		
Mirrors and Reading Aids	✓		✓										2	✓	✓	✓	✓
Filtered Glasses	✓		✓										2	✓	✓	✓	✓
Reading Glasses	✓												1	✓	✓	✓	✓
Talking Calculators	✓		✓										2	✓	✓	✓	✓
Key Boards/ Covers	✓		✓										2	✓	✓	✓	✓
Talking Tools	✓		✓										2	✓	✓	✓	✓
Tactile Measuring Tools	✓		✓										2	✓	✓	✓	✓
Tactile Rulers	✓		✓										2	✓	✓	✓	✓
Phones	✓		✓										2	✓	✓	✓	✓
Clocks	✓		✓										2	✓	✓	✓	✓
Large-Face Watches	✓		✓										2	✓	✓	✓	✓
Tactile Watches	✓		✓										2	✓	✓	✓	✓
Talking Watches	✓		✓										2	✓	✓	✓	✓
Braille Watches													0	✓		✓	
Eye Drop Aids	✓		✓										2	✓	✓	✓	✓
Talking Scale	✓		✓										2	✓	✓	✓	✓
Pill Boxes	✓		✓										2	✓	✓	✓	✓
Medical Devices													0	✓	✓	✓	✓
Talking Thermometer	✓		✓										2	✓	✓	✓	✓
Canes/Walking Sticks			✓										1	✓	✓	✓	✓
Mobility Accessories			✓										1	✓	✓	✓	✓
Kitchen Utinsels	✓		✓										2	✓	✓	✓	✓
Sewing Tools	✓		✓										2	✓	✓	✓	
Games	✓		✓										2	✓	✓	✓	✓
Funded By	HSE, Fundraising, Donations, Retail Shops	Prison Service	Department of Social Protection	HSE, Department of Education and Skills, Fundraising, Events, Donations	Fundraising, Corporate Support	Donations, Fundraising, HSE		HSE, Fundraising, Donations	Fundraising, Donations	HSE	Department of Education and Skills	ICS Membership Dues, Skills Courses					
